# Low Folate Levels are Associated with Strokes and Less Venous Thromboses in Primary Antiphospholipid Syndrome

**DOI:** 10.31138/mjr.33.4.478

**Published:** 2022-11-23

**Authors:** Jozélio Freire Carvalho, Yehuda Shoenfeld

**Affiliations:** 1 Institute for Health Sciences from Federal University of Bahia, Salvador, BA, Brazil,; 2 Zabludowicz Research Centre for Autoimmune Diseases, Sheba Medical Centre, Israel

**Keywords:** antiphospholipid syndrome, thrombophilia, folic acid, vitamin, stroke

## To the editor,

Antiphospholipid syndrome (APS) is associated with venous and/or arterial thromboses, recurrent miscarriages, and/or thrombocytopenia.^1^ In addition, it is known that some patients may develop cardiovascular events linked to traditional and non-traditional risk factors.^2^ Low folate levels have been associated with comorbidities, including strokes.^3^ However, we were unable to find any previous study which has evaluated the clinical and laboratory associations of FL in primary APS patients. Therefore, it would be reasonable to evaluate if serum folate levels (SFL) might be connected to clinical (strokes) or laboratory features of APS, independently of homocysteine and vitamin B12 levels.

A sample of 23 primary APS (pAPS) was studied to verify clinical and serological differences between individuals with normal and low SFL. Twenty-three pAPS females (Sidney criteria)^4^ were enrolled for this cross-sectional analysis, which excluded individuals with associated connective tissue disease and using folate supplementation. Data were assessed by a chart review and clinical examinations. IgG and IgM anticardiolipin antibodies (ACL) were tested at least twice using ELISA (Inova Diagnostics, Inc., San Diego, USA). In addition, lupus anticoagulant (LAC) was determined according to the international guidelines.^5^ Serum folate levels were measured in a Beckman-Coulter DxI 800 chemiluminescent immunoassay, and normal levels were considered above 6.8 ng/mL.^6^

In this sample, 4/23 (17.4%) have had low SFL (group 1), and 19/23 (82.6%) did not (group 2). Median SFL between the groups were significantly different (4.85 [3.5–5.8 ng/mL] vs. 12.1 [7.4–20 ng/mL], p=0.0003). Group 1 patients were older (50 [42–52 years] vs. 40 [21–54 years], p=0.04), had more Sneddon’s syndrome (75% vs. 21%, p=0.04), strokes (75% vs. 26.3$, p=0.05), live-do reticularis (75% vs. 21%, p=0.02), acute myocardial infarction (25% vs. 0, p=0.01), family history of coronary diseases (50% vs. 5.2%, p=0.03), and currently were under low molecular weight heparin (75% vs. 26.3%, p=0.03) in comparison with group 2. On the other hand, group 1 had less venous thromboses event (0 vs. 47.3%, p=0.01), and consumed less glucocorticoid (0 vs. 58%, p=0.017) than group 2. No other variable was significantly different between groups 1 and 2, including homocysteine and vitamin B12 levels (p> 0.05). We proceeded a logistic regression analysis, and the independent variables associated with low folate levels were stroke (p=0.03), and less venous thrombosis (p=0.02).

The present study found for the first time a positive association of low folate levels with strokes, and a negative association with venous thromboses in patients with pAPS. Previous studies of the literature have shown a positive link between low folate levels and stroke in hypertensive patients.^3^ The risk of first ischemic stroke was significantly higher in hypertensive patients with low levels of both folate and B_1,_ and the authors suggesting a folic acid supplementation for this risk population. Furthermore, a meta-analysis with 55,764 subjects studied concluded that folic acid supplementation may prevent stroke.^7^ It is logical to think that low folate can induce hyper-homocysteine, although we did not find this typical association in the present study. One hypothesis is that low folate creates an inflammatory environment due to low methylation. It is well known that folic acid has a direct antioxidant, antithrombotic, and endothelium-protective effects and has a significant role in DNA synthesis or repair.^8^ Ultimately might induce endothelial damage, and a patient may become prone to stroke. This study has the great advantage of including pAPS patients exclusively, excluding other connective tissue diseases, avoiding the consequences of associated diseases. However, a limitation of the present study is the relatively low number of participants, and it might bring type 1 errors in the interpretation of the results.

In conclusion, 17% of the PAPS patients had low folate levels. Serum folate levels in pAPS is linked to stroke and less venous events. In patients with folate levels below the normal range, we recommend supplementing folic acid for these APS subjects.
